# The impact of protected areas on poverty: evidence from Chile

**DOI:** 10.1186/s40693-022-00110-0

**Published:** 2022-06-28

**Authors:** Thais Vilela, Alfonso Malky Harb, Carla Mendizábal Vergara

**Affiliations:** 1 Conservation Strategy Fund, Arcata, CA USA; 2 Conservation Strategy Fund, La Paz, Bolivia

**Keywords:** Protected areas, Poverty, Chile, Patagonia, Differences-in-Differences, Q5, Q010, Q560, O13

## Abstract

In this paper, we estimate the causal relationship between protected areas and poverty in Chile from 1982 to 2002. Chile is part of the coalition of countries committed to protecting 30% of the planet by 2030; a decision that implies increasing the number of protected areas in the country. As a result of this decision, grows the national debate about the potential impacts of protected areas on the economy and society. By estimating the causal effect of protected areas on poverty, we aim to contribute to this debate in Chile. We use panel data and a quasi-experimental approach to estimate the causal effect. We find that establishing a protected area covering at least 17% of a unit’s terrestrial area causes a reduction of 0.216 standard deviations in the poverty index. This result is not sensitive to arbitrary implementation choices. Additionally, we show that the effect is driven by the Patagonia region, the part of Chile with the largest amount of new protected areas during the time frame of this study. Besides showing the benefits of protected areas to society, we hope the findings presented here might also be used to attract new investments and financial support to protected areas currently underfunded in Chile.

**Supplementary Information:**

The online version contains supplementary material available at 10.1186/s40693-022-00110-0.

## Background

There is an active debate in academia about whether protected areas reduce or increase poverty [1–4]. Protected areas can provide ecosystem services, promote tourism and other recreational activities, and improve infrastructure in remote areas [5–8]. These potential benefits can lead to higher income and better quality of life for disadvantaged communities around those areas. But the establishment of protected areas is also associated with forced eviction, restriction to local communities' use of natural resources, and other limitations to extractive activities [9–12]. The possibility of these negative effects has fueled opposition to protected areas in countries as varied as Australia, Germany, Thailand, and the United States [13, 14].

Given Chilean support for the global effort to protect at least 30% of the planet by 2030, this debate is particularly relevant in the country. Still, the answer to this debate is far from clear-cut: research surveys document that communities surrounding protected areas view these areas as both improving and damaging well-being (measured not only through poverty levels but also through other socioeconomic variables) [15–17]. Several studies—done for countries other than Chile—find a correlation between the presence of protected areas and high poverty levels [18, 19]. But these studies failed to demonstrate a causal link between these two variables [20]. They may instead capture the fact that protected areas are usually established in areas with high poverty levels. Other studies find poverty-reducing effects of protected areas in other countries, but it is not clear whether those results can be extrapolated to the Chilean context [21–23].

Additionally to the empirical debate, there is also an ongoing theoretical discussion about the link between protected areas and poverty alleviation. The debate is more focused on the purpose of protected areas. On the one hand, some conservationists believe that protected areas should be used to conserve nature and should not be required to provide socioeconomic benefits to society – at least not directly [24]. On the other hand, some believe that protected areas and poverty are intertwined. And, as a result of this situation, protected areas should not only conserve nature but also be used as an instrument to contribute to the well-being of local communities (including poverty alleviation) [25]. In between these two arguments, there is the belief that protected areas should be used primarily to conserve nature, but efforts should be made by governments and international and national non-governmental institutions to guarantee that local communities are not negatively (and disproportionally) affected by the establishment of protected areas [26].

The existence of this mixed evidence in the literature and the ongoing debate suggest that more research is needed on this topic. In this paper, we contribute to the empirical literature by estimating the causal relationship between protected areas and poverty in Chile. We employ a differences-in-differences econometric design using Census data from 1982 to 2002. This time frame was defined based on the availability of data. Our dependent variable is a poverty index based on questions such as whether the household has access to electricity, piped water, and a refrigerator. Poverty is understood here as a multi-dimensional variable instead of a measure of income. Specifically about the econometric approach, the differences-in-differences design is more credible than a cross-sectional comparison of locations because it nets out the effect of intrinsic characteristics that might correlate with protected areas, even if these characteristics are not observed by the researcher (e.g., protected areas might be located in areas with limited infrastructure, soil quality that is less suitable for agriculture, or with worse schools). It is also better than time-series analysis—without a control group—because it allows for trends that are unrelated to protected areas, such as decreased poverty over time due to higher schooling achievement and economic growth.

In addition to contributing to the empirical debate, we hope this type of study can assist institutions such as CONAF (Chilean’s National Forest Corporation) to make the case for protected areas and support more funding from national governments. The underline assumption is that if we are able to show that protected areas help to alleviate poverty, then decision-makers might feel that supporting protected areas is a wise investment as they conserve nature and positively impact local communities. Currently, Chile is among the ten most underfunded countries for biodiversity conservation in the world [27] and only 12% of protected areas in Chile are effectively managed [28, 29].

## Methods

**Fig. 1:**
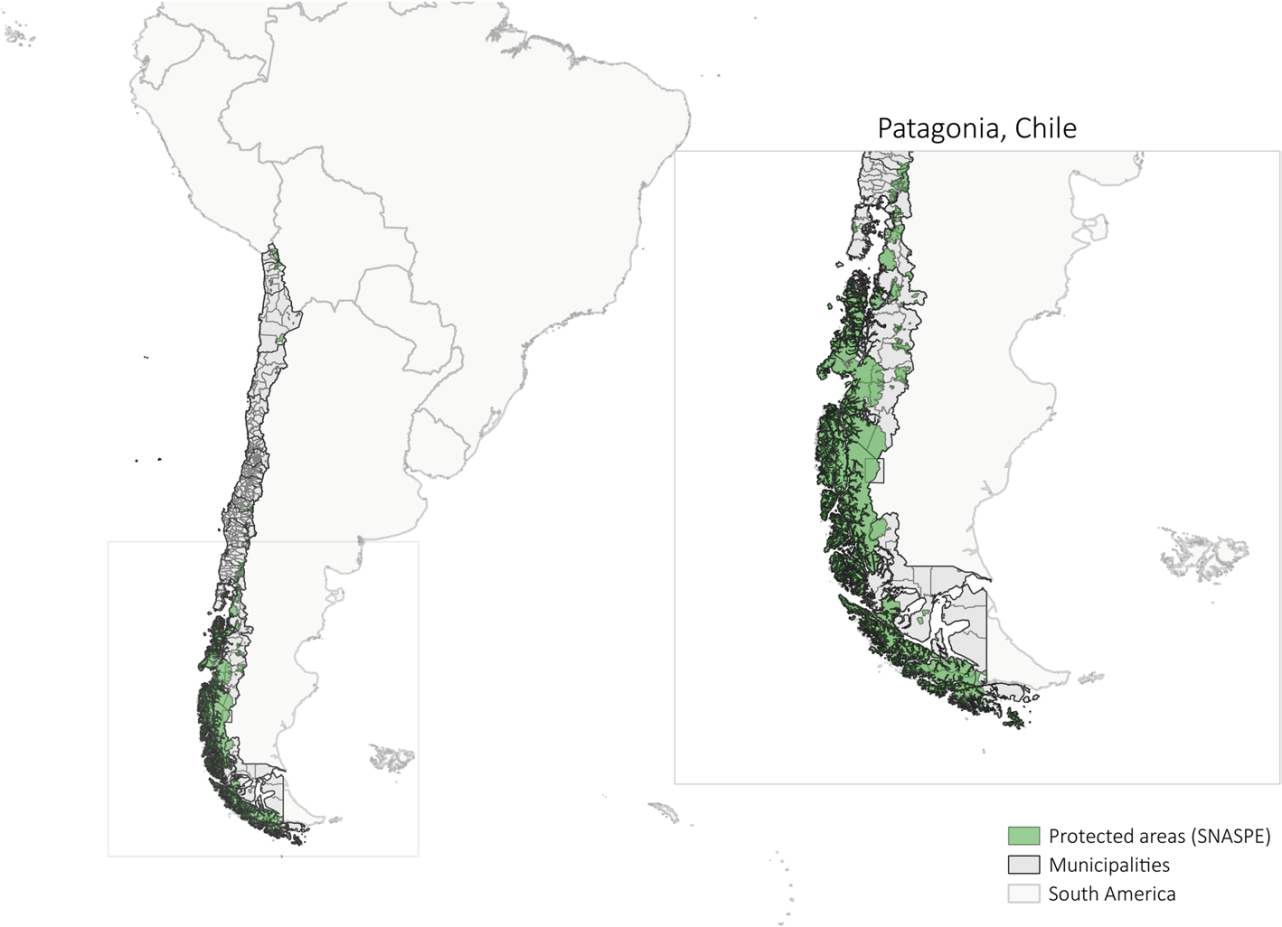
Terrestrial protected areas in Chile. To the best of our knowledge, there is no formal geographic definition of Patagonia in Chile. In this study, we define Chilean Patagonia as the area that encompasses the regions Los Lagos, Aysén del General Carlos Ibáñez del Campo, and Magallanes and Chilean Antarctica. This geographic delimitation was based on personal communication with researchers in Chile. SNASPE is the acronym in Spanish for the national state system of protected areas managed by the *Corporación Nacional Forestal* (CONAF). It is composed of the following categories National Parks, National Reserves, and Natural Monuments. Natural Sanctuaries, another terrestrial protected area category, are not part of the SNASPE and were not included because of their limited total surface, most of them are recently created and their administration is limited in comparison to the SNASPE. See Table S3 in the Additional Material file for the complete list of the protected areas considered in this study as well as the year these protected areas were created by the State. The analysis focused only on terrestrial protected areas from SNASPE

Protected areas in Chile cover approximately 20% of the land and about 40% of the marine total Chilean territory. Out of the protected land territory, about 87% is located in the Patagonia region (Fig. 1 and Table S1 in the Additional Material file), an important wilderness area in the southern hemisphere [30]. Since 1990, poverty has been decreasing in Chile [31]. The last survey on poverty conducted (without the impacts of the Coronavirus) by the Chilean government in 2017—named Casen 2017—shows a reduction of about 35 and 18 percentage points between 2006 and 2017 in the rural and urban areas respectively [32, 33]. However, despite the importance of this survey, the Patagonia region is not well represented in it due to the difficulty in accessing municipalities located in Patagonia, some important areas are excluded from CASEN (Table S2 in the Additional Material file). Because of the importance of the Patagonia region for our research in relation to protected areas, we create our own poverty index using Census data.

The data is from Chile’s National Statistics Institute (INE is the acronym in Spanish), but we obtained it from IPUMS-International [34]. Throughout the years, there were changes in the methodology used to conduct the census surveys, as well as boundary changes. IPUMS adjusts for these changes making it easier to conduct the analysis. At the time of this study, the data at IPUMS were available for the years 1982, 1992, and 2002 – which correspond to the time period of this study. The unit of analysis is called geolev2. The data is organized at the third major administrative unit—in Chile’s case, Municipalities. However, to account for boundary changes across census years, the variables are spatially harmonized by IPUMS. In total, we have 178 units in each year.

Table 1 shows the five household characteristics from the census surveys used to create the poverty index. We tested some additional common variables traditionally used in the literature to measure poverty, such as access to hot water, computer, and automobiles, but decided not to use them because of the large amount of missing data (especially for the year 1982) and consequently loss of observation.

**Table 1:** Household characteristics used in the poverty index

Household characteristics	Poverty indicator
Has electricity?	No
Has refrigerator?	No
Has piped water?	No
Connected to the sewage system?	No
Toilet facilities	Non-flush (e.g., latrine) or no toilet

Once the households’ characteristics to be used in the poverty index are defined, we use the Principal Component Method to combine them and create the index [35]. This method reduces the dimensionality of the data set by combining the variables linearly. The resulting variables from the linear combinations are named components and store the variation in the dataset. The first component has the highest variation (highest information value) while the last component has the lowest variation. In this study, we define the poverty index as the first component. In our case, this component accounts for 64% of the whole variance in the data set. Additionally, the analysis of the first component shows that the variables follow the same direction and are equally important. Having said that, the poorest units are the ones in which households, on average, lack basic needs such as electricity and piped water (Table 1). The higher the index, the poorer the unit.

Because the poverty unit has no natural metric, its interpretation is not obvious. To overcome this, we standardized the variable by subtracting the mean from every observation and then dividing them by the sample standard deviation. Interpretations now are done in terms of one standard deviation (which is equivalent to 1.39 in the original poverty measure).

### Econometric model

In this study, protected areas are natural monuments, national parks, and national reserves. These are the areas managed by SNASPE. To establish the impact of protected areas on poverty, we use the differences-in-differences approach. This method evaluates the impact of an intervention by comparing the changes in the outcome of interest over time and between two groups: the treated group and the control group.

The intervention is defined here as having at least 17% of the terrestrial unit protected. This threshold is based on the Convention on Biological Diversity (CBD) Aichi Biodiversity Target to protect at least 17% of land and 10% of oceans by 2020. The definition of the threshold is based on [36] but the percentage used here is different to account for an update in the target. We conduct some sensitivity analysis by changing this threshold to 10 and 30% in other specifications. The higher threshold mirrors a proposal by CBD to protect 30% of the planet by 2030 [37].

Based on the definition of the intervention, we define the treated group as units with at least 17% of their terrestrial area protected. The control group consists of units with less than the threshold protected. Under these two definitions, the units in the treated and control groups are not necessarily the same for the three periods (1982, 1992, and 2002). For example, in the control group, besides the units with less than 17% of their terrestrial area protected throughout the time frame of this study, we also have units that initially had a lower percentage of their area protected but that, at some point in time, for example, between 1993 and 2001, had an increase in the percentage protected to or above 17%. This means that for the years 1982 and 1992, these units would be in the control group, but for 2002 they would be part of the treated group.

To obtain the causal effect, the differences-in-differences estimator compares the average change in poverty in the treated units to the average change in the control units, for the same period. This identification strategy is implemented through a panel regression model with unit fixed effects and time fixed effects. The key identifying assumption is that poverty index trends would be the same in all units in the absence of a new protected area [38].

Let ${y}_{it}$ be the poverty index in unity $i$ and year $t$. The model of interest is:

1 $${y}_{it }= {a}_{0 }{D}_{it }+{{a}_{1 }{x}_{it }+a}_{2i}+{a}_{3t}+{e}_{it}$$

where ${D}_{it}$ is a binary variable equal to one in the case the unit has at least 17% of its area protected in year $t$ and zero otherwise. The estimated coefficient on the ${D}_{it}$ variable recovers the average treatment effect on the treated units.

The time-varying control ${x}_{it}$ is the log of the population size at unit $i$ in year $t$, intended to capture other unit-specific events leading to socioeconomic development [39]. Including this control is a conservative approach. If protected areas cause changes in population that are concomitant to changes in poverty, part of that causal effect might be absorbed by this control. We include it to increase the likelihood that, if a statistically significant effect between protected areas and poverty is recovered, it reflects a causal relationship.

Given that most protected areas in Chile are located in Patagonia, one might wonder whether protected areas in that region have a different impact on poverty when compared to the impact of protected areas in other regions of Chile. To test this hypothesis, we run an alternative model in which we interact the binary variable of interest, ${D}_{it}$, with a binary variable, named Patagonia, equal to 1 if the unit is located in Patagonia and 0 otherwise. Here we define Chilean Patagonia as the area going from *Los Lagos* region (municipalities *Puyehue*, *San Pablo*, and *San Juan de la Costa*) to *Mangallanes y Antártica Chilena* region.

As a final step and to obtain more information on the importance of protected areas to reduce poverty, we change the explanatory variable of interest to the fraction of the area that is protected in each unit. Besides avoiding the need for us to have a threshold, this change allows us to estimate the variation in the poverty index given a 1% change in the amount protected. As with the main model, we interact this new variable with the location of the unit to assess the difference between the impact of protected areas in and outside the Patagonia region. However, different from the main model, this alternative specification assumes a linear relationship between protected areas and poverty. In other words, it assumes that the effect of protected areas on poverty is proportional to the protected areas’ share in each unit.

## Results

**Fig. 2:**
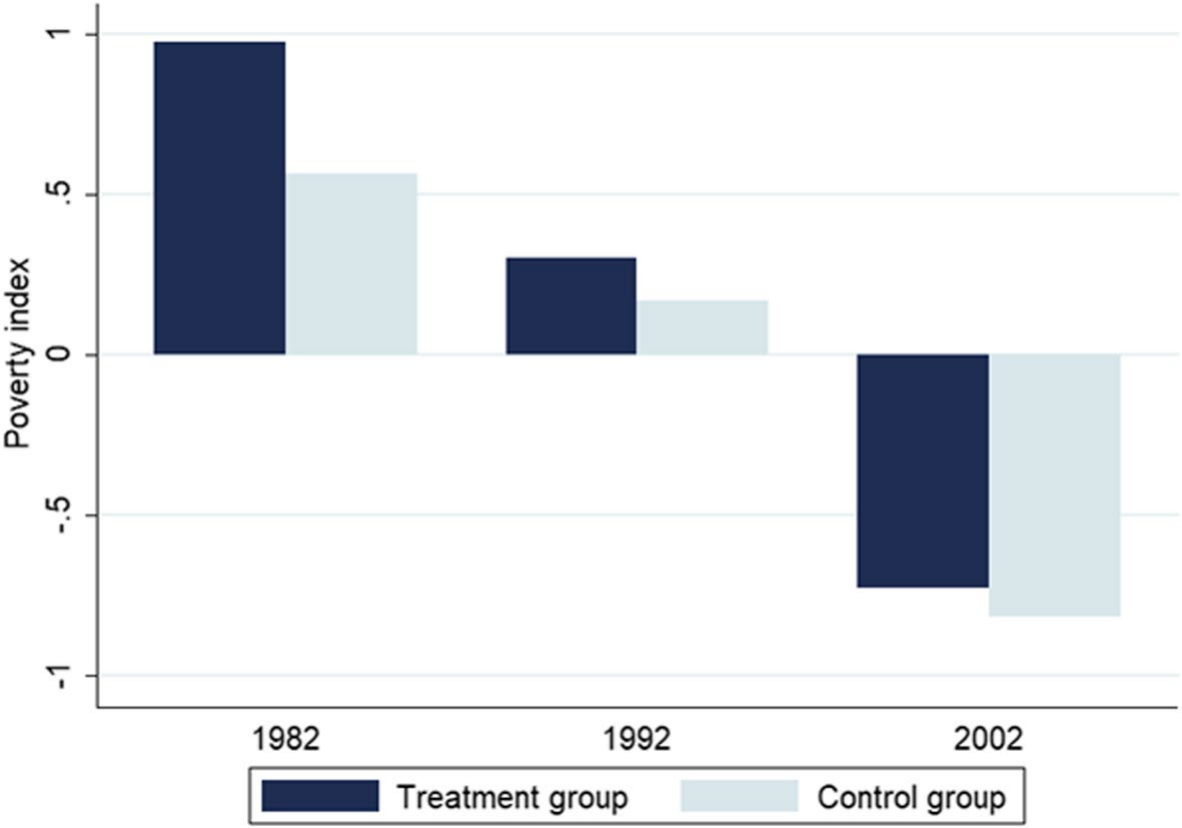
Average poverty index by year and group. Notes: The treated group consists of units with at least 17% of their terrestrial area protected. The control group consists of units with less than the threshold protected

Similar to other researchers that studied the relationship between protected areas and poverty [22, 33], we find that the average poverty in the treated group (with at least 17% of the terrestrial area protected) is higher than in the control group. Figure 2 shows that the poverty index is 73%, 79%, and 33% higher in 1982, 1992, and 2002, respectively, in administrative units with a protected area. Although this result implies a positive relationship between both variables of interest, it does not mean causation. The identified correlation shows simply that protected areas and poverty moved in the same direction from 1982 to 2002, but it does not imply that protected areas led to a change in the poverty index. Other factors (e.g., location of protected areas) might affect both variables and bias the estimation.

Based on the identification strategy described above, we are able to estimate the causal effect of protected areas on poverty in Chile. Table 2 shows the main results. Column 1 shows that in units with at least 17% of their area protected, the poverty index is 0.216 standard deviations lower than units with less than 17% of their area protected. This result is significant at the 0.1 level and robust to two different thresholds, 10 and 30% (Columns 2 and 3, respectively). Column 4 shows the results for the model in which we have an interaction between the binary variable of interest and the location of the units (if in Patagonia or not). We find that the treatment effect in Patagonia is substantially stronger, and this result is statistically significant at the 0.01 level. Once we add the interaction variable to the model, the treatment effect in areas outside Patagonia falls to only 0.05 standard deviations, and it is statistically indistinguishable from zero. This result suggests that protected areas have sizable poverty-reducing effects, but only in Patagonia.

**Table 2:** Estimated effects of protected areas on poverty in Chile. The main explanatory variable is a binary variable equal to 1 if the unit has at least a fraction of its area protected

	Outcome of interest: poverty index			
	Main model	Alternative model specifications		
	(1)	(2)	(3)	(4)
Binary variable = 1 if at least 17% is protected	-0.216* (0.127)			-0.050 (0.138)
Binary variable = 1 if at least 10% is protected		-0.229* (0.118)		
Binary variable = 1 if at least 30% is protected			-0.220 (0.131)	
${D}_{it}$ * Patagonia, where ${D}_{it}$ is a binary variable = 1 if at least 17% is protected				-0.580** (0.205)
$log(population)$	0.265 (0.194)	0.261 (0.194)	0.263 (0.194)	0.263 (0.192)
Constant	-3.474 (2.311)	-3.470 (2.275)	-3.491 (2.282)	-3.488 (2.255)
Unit-fixed effect	Yes	Yes	Yes	Yes
Year-fixed effect	Yes	Yes	Yes	Yes
No. of observations	534	534	534	534
${R}^{2}$	0.914	0.914	0.914	0.916

Robust standard errors clustered at the unit level are reported in parentheses. The levels of significance are *10%, and **1%

While we do not have a conclusive answer for what explains that heterogeneity. One hypothesis is that the Patagonia region is poorer, and protected areas have a larger impact on poorer areas. Another possible explanation is that protected areas in Patagonia are intrinsically different from the ones in other areas. More research is needed to investigate the source of that disparity. Table S4 in the Additional Material file shows additional robustness results.

Table 3 shows the results considering the faction of the unit’s area that is protected as the explanatory variable of interest. Column 1 shows that a 1% increase in the fraction of the area that is protected reduces the poverty index in 0.01 standard deviations. Although the direction of the response is negative and the magnitudes align well with our preferred specification, the coefficient is not statistically significant at the 0.1 level. Column 2 also shows that we are not able to reject the null hypothesis that protected areas do not affect poverty at the 0.1. level. Additionally, under this model, the coefficient associated with the treatment effect in Patagonia is not statistically significant. Further research is needed to better understand the different results obtained under the main model and this alternative specification. A possible explanation relates to the strong assumption of linearity between protected areas’ size and poverty in the alternative model.

**Table 3:** Estimated effects of protected areas on poverty in Chile. The main explanatory variable is a continuous variable equal to the fraction of the unit’s area that is protected

	Outcome of interest: poverty index	
	Main model under new specification	Alternative model under new specification
	(1)	(2)
Fraction of the unit’s area protected	-0.010 (0.006)	0.009 (0.017)
Fraction of the unit’s area protected Patagonia		-0.024 (0.018)
$log(population)$	0.259 (0.196)	0.259 (0.197)
Constant	-3.432 (2.305)	-3.431 (2.309)
Unit-fixed effect	Yes	Yes
Year-fixed effect	Yes	Yes
No. of observations	534	534
${R}^{2}$	0.913	0.914

Robust standard errors clustered at the unit level are reported in parentheses

## Discussion

Our findings corroborate other studies in the literature in two main aspects. First, we contribute to the empirical evidence that a positive correlation exists between protected areas and poverty. The simple comparison between the poverty index in treated units and the units in the control group shows that units in the treated group are poorer. Indeed, almost 90% of all protected areas are located in the Patagonia region – the poorest region in Chile [40]. Second, using a quasi-experimental approach, we show that protected areas alleviate poverty. Other studies have found similar results for other countries. However, comparisons among the magnitude of the causal effects found in the literature are difficult as the metric used to define and quantify poverty tends to be different in each study.

In terms of future research, we highlight two. First, the need to better understand the reasons why the results found in this study are driven by the Patagonia region. Second, the importance to identify the channels through which protected areas affect poverty in Chile. Below we provide an initial discussion about the second point based on the literature on this topic.

In Costa Rica, for example, tourism and recreational services account for nearly two-thirds of the total poverty reduction that comes from the establishment of protected areas. The remaining percentage is due to improvements in infrastructure and other ecosystem services besides tourism [7].

In the Chilean case, tourism seems to play an important role too. In 2019, nearly 3,5 million people visited at least one protected area in Chile [41]. This number is about 78% of the total number of tourists that have visited Chile in the same year [42]. The income generated by tourists—one of the main factors contributing to poverty alleviation—has increased about 7.3% per year for ten years, from 2006 to 2016 [43].

In the Patagonia region – the area with the highest number of protected areas—the number of tourists in 2018 was approximately 420,000 people [44]. This total, however, might be underestimated since no data is available for all protected areas in the region (for example, Pumalín Douglas Tompkins National Park). We also note that the number of tourists visiting protected areas varies considerably. For example, Torres del Paine National Park accounts for 69% of Patagonia’s protected areas. This discrepancy might result in unequal benefit distribution among populations living nearby protected areas. To this point, a strong social and political national and regional framework is equally important to guarantee that protected areas successfully contribute to poverty reduction [45].

Another possible channel through which protected areas might reduce local poverty is via infrastructure [7, 35]. To encourage tourism to remote places national and regional governments tend to improve local infrastructures such as roads, and transmission lines. Although we do not have data on the differences between connectivity in Patagonia and outside, the literature shows that Chile has good connectivity. For example, in terms of road connectivity, measured as the average speed and straightness of an itinerary connecting the 10 or more largest cities, Chile ranks as one the best countries in Latin America and the Caribbean [46]. In terms of connectivity between the south region (Patagonia) and the rest of the country, “the main vertical north–south highway (Route 5) and most transversal arteries linking the key cities have already been built” [47].

Ideally, we would like to control for road improvement throughout the years in our model. However, data on road networks were not found for the years 1982, 1992, and 2002. By not including this confounding variable—connectivity—the effects associated with the protected area variable could be biased if we assume that the connectivity has improved mostly in areas with new protected areas. The direction of the bias, however, is not trivial. On the one hand, roads reduce transportation costs and travel time. These reductions tend to increase mobility and improve job opportunities, reducing poverty in the region. On the other hand, roads increase competitiveness. As a result, local (and smaller) producers might suffer from cheaper national and international products. If this is the case, the effect on poverty would be positive (i.e., poverty would increase). More research is needed to understand the impact of infrastructure on the local economy—especially in initially isolated places.

### Limitations of the study

This study has some potential limitations. First, the poverty index includes only a subset of the most common variables used to create a multi-dimensional poverty measure. In the case of this study, all variables included are related to the standard of living (for example, electricity and sanitation). As a result, we are not capturing the impact of protected areas on health and education—two dimensions traditionally considered when creating a poverty index. Second, the sample size is not very large, with only three time periods. That limitation affects the degree of precision of the estimated causal effects. Third, while we find that results are driven by protected areas in the Patagonia region drive results, we do not have enough information in the data set to understand this result.

## Conclusions

This paper estimates the causal relationship between protected areas and poverty in Chile. We employ a differences-in-differences econometric design using Census data from 1982 to 2002. We find that protected areas reduce poverty—measured as a multi-dimensional index. Using our preferred specification, we show that establishing a protected area covering at least 17% of the territorial administrative unit causes a reduction of 0.216 standard deviations in the poverty index. We highlight that this effect comes from the Patagonia region, the part of Chile with the largest amount of new protected areas. Further research is needed to better understand and quantify all possible mechanisms through which protected areas reduce poverty in Chile, as well as to adequately address all possible confound variables (e.g., infrastructure) that otherwise might bias the results. Data limitation might be a bottleneck in Chile, being important for government institutions to invest in future data collection.

In addition to contributing to the literature, we hope that government institutions in Chile could also use the results from this study to justify increasing the national budget for protected areas. Our study provides evidence that protected areas might be a wise investment for both economic and social development. At the time of writing this study, there was an active discussion in Chile about a new Constitution. Among the topics being discussed, we highlight the importance of protected areas to Chileans and possible mechanisms to fund these conservation and recreational areas.

## Supplementary Information

**Additional file 1:** **Table S1.** Municipalities located in Patagonia. **Table S2.** Areas excluded from CASEN 2017. **Table S3.** Protected areas by IPUMS units. **Table S4.** Additional robustness estimations. Estimated effects of protected areas on poverty in Chile. The main explanatory variable is a binary variable equal to 1 if the unit has at least a fraction of its area protected.

## Data Availability

The data that support the findings of this study are publicly available from IPUMS Internationals and CONAF. In the Additional Material file, we include some supporting information based on data collected from IPUMS and CONAF.
