# Supplementary material for: The impact of protected areas on poverty: evidence from Chile
Source: Rev. Chil. de Hist. Nat. 2022 Jun 28;95(1):5. doi: 10.1186/s40693-022-00110-0 (PMC9243912; doi:10.1186/s40693-022-00110-0)
Supplement: Supplementary file 1 — Additional file 1: Table S1. Municipalities located in Patagonia. Table S2. Areas excluded from CASEN 2017. Table S3. Protected areas by IPUMS units. Table S4. Additional robustness estimations. Estimated effects of protected areas on poverty in Chile. The main explanatory variable is a binary variable equal to 1 if the unit has at least a fraction of its area protected. [file 40693_2022_110_MOESM1_ESM.docx]

**Additional Material**

**The impact of protected areas on poverty: Evidence from Chile**

Thais Vilela^a^, Alfonso Malky Harb^b^, and Carla Mendizábal Vergara^b^

^a^ Conservation Strategy Fund, Arcata, CA 95521.

^b^ Conservation Strategy Fund, La Paz, Bolivia.

This document contains: Tables S1 – S4

**Additional Material**

Table S1: Municipalities located in Patagonia.

| **Municipalities with protected areas** | **Municipalities without protected areas** |
| --- | --- |
| Ancud, Aysén, Cabo de Hornos, Castro, Chaitén, Chile Chico, Chonchi, Cisnes, Cochamo, Cochrane,  Coyhaique, Dalcahue, Futaleufú, Hualaihué, Lago Verde, Natales, O'Higgins, Palena, Porvenir, Puerto Montt, Puerto Octay, Puerto Varas, Punta Arenas, Puyehue, Río Ibáñez, Río Verde, San Gregorio, Timaukel, Torres del Paine, and Tortel. | Calbuco, Curaco de Vélez, Fresia, Frutillar, Guaitecas, Laguna Blanca, Llanquihue, Los Muermos, Maullin, Osorno, Primavera, Puqueldon, Purranque, Queilen, Quellon, Quemchi, Quinchao, Río Negro, San Juan de la Costa, and San Pablo. |

Note: This table shows the municipalities that have protected areas. We do not impose any threshold here.

Table S2: Areas excluded from CASEN 2017.

| Areas excluded from the CASEN survey | General Lagos, Colchane, Ollagüe, Juan Fernández, Isla de Pascua, **Cochamó**, **Chaitén**, **Futaleufú**, **Hualaihué**, **Palena**, **Lago Verde**, **Guaitecas**, **O’Higgins**, **Tortel**, **Laguna Blanca**, **Río Verde**, **San Gregorio**, **Cabo de Hornos (Ex - Navarino)**, Antártica, **Primavera**, **Timaukel**, **Torres del Paine.** |
| --- | --- |

Note: All municipalities in bold are located in the Patagonia region.

Table S3: Protected areas by IPUMS units.

| **Name of the Protected Areas** | **Protected Areas Establishment Decree** | **Year of establishment** | **IPUMS unit (geolev2)** |
| --- | --- | --- | --- |
| Monumento Natural Canquen Colorado | N41 04102017 MMA | 2017 | 152121001 |
| Monumento Natural Cerro Ñielol | N617 03121987 MBN | 1987 | 152091001 |
| Monumento Natural Cinco Hermanas | N160 13101982 MA | 1982 | 152112001 |
| Monumento Natural Contulmo | N160 12101982 MA | 1982 | 152082002 |
| Monumento Natural Cueva Del Milodon | N359 16121993 MA | 1993 | 152124001 |
| Monumento Natural Dos Lagunas | N160 13101982 MA | 1982 | 152111001 |
| Monumento Natural El Morado | N162 199774 MA - N121 04122017 | 1974 | 152132002 |
| Monumento Natural Isla de Cachagua | N89 270689 MA | 1989 | 152054001 |
| Monumento Natural Islote de Puñihuil | N130 29091999 MA | 1999 | 152102002 |
| Monumento Natural La Portada | N51 030490 MA | 1990 | 152021001 |
| Monumento Natural Laguna De Los Cisnes | N160 13101982 MA | 1982 | 152121001 |
| Monumento Natural Lahuen Ñadi | N14 10012000 MA | 2000 | 152101001 |
| Monumento Natural Los Pingüinos | N160 13101982 MA | 1982 | 152121001 |
| Monumento Natural Paposo Norte | N7 030214 MMA | 2013 | 152021001 |
| Monumento Natural Picaflor de Arica | N91 25102018 MMA | 2018 | 152012001 |
| Monumento Natural Pichasca | N123 171085 MA | 1985 | 152043002 |
| Monumento Natural Quebrada De Cardones | N64 010709 MBN | 2009 | 152012001 |
| Monumento Natural Salar De Surire | N29 080383 MA | 1983 | 152012001 |
| Parque Nacional Alberto De Agostini | N136 24041985 MBN | 1985 | 152121001 |
| Parque Nacional Alerce Andino | N735 17111983 MBN | 1983 | 152101003 |
| Parque Nacional Alerce Costero | N9 07052010 MBN - N60 07062013 MBN | 2010 | 152105002 |
| Parque Nacional Archipielago Juan Fernandez | N606 160889 MBN | 1989 | 152051002 |
| Parque Nacional Bernardo OHiggins | N392 14061989 MBN | 1989 | 152124001 |
| Parque Nacional Cabo De Hornos | N995 26041945 MT | 1945 | 152121001 |
| Parque Nacional Cerro Castillo | N88 27072017 MBN | 2017 | 152111001 |
| Parque Nacional Chiloé | N368 27122000 MBN | 2000 | 152102001 |
| Parque Nacional Conguillio | N131 15041987 MBN | 1987 | 152091005 |
| Parque Nacional Corcovado | N4 06012018 MBN | 2018 | 152112001 |
| Parque Nacional Fray Jorge | N867 301281 MBN | 1981 | 152043001 |
| Parque Nacional Hornopirén | N2 15012018 MBN | 2018 | 152101003 |
| Parque Nacional Huerquehue | N82 02031985 MBN | 1985 | 152091012 |
| Parque Nacional Isla Guamblin | N321 01061967 MA | 1967 | 152112001 |
| Parque Nacional Isla Magdalena | N101 27092017 MBN | 2017 | 152112001 |
| Parque Nacional Kawésqar | N6 26012018 MBN | 2018 | 152121001 |
| Parque Nacional La Campana | N228 140685 MBN | 1985 | 152055004 |
| Parque Nacional Laguna Del Laja | N652 250658 MA | 1958 | 152083004 |
| Parque Nacional Laguna San Rafael | N737 30111983 MBN | 1983 | 152112001 |
| Parque Nacional Lauca | N29 080383 MA | 1983 | 152012001 |
| Parque Nacional Llanos De Challe | N946 290794 MBN | 1994 | 152033001 |
| Parque Nacional Llullaillaco | N856 030895 MBN | 1995 | 152021001 |
| Parque Nacional Melimoyu | N5 26012018 MBN | 2018 | 152112001 |
| Parque Nacional Morro Moreno | N5 28012010 MBN | 2010 | 152021001 |
| Parque Nacional Nevado Tres Cruces | N947 280794 MBN | 1994 | 152031001 |
| Parque Nacional Pali-Aike | N900 21071994 MBN | 1994 | 152121001 |
| Parque Nacional Palmas De Cocalán | N26 11011989 MA | 1989 | 152135001 |
| Parque Nacional Pan De Azúcar | N527 071085 MBN | 1985 | 152021001 |
| Parque Nacional Patagonia | N98 25102018 MBN | 2018 | 152112001 |
| Parque Nacional Pumalin | N28 28022018 MBN | 2018 | 152101003 |
| Parque Nacional Puyehue | N145 21122017 MBN | 2017 | 152101003 |
| Parque Nacional Queulat | N640 13101983 MBN | 1983 | 152112001 |
| Parque Nacional Radal Siete Tazas | N15 27032008 MNB | 2008 | 152073002 |
| Parque Nacional Rapa Nui | N119 27112017 MNB | 2017 | 152051002 |
| Parque Nacional Tolhuaca | N28 23011985 MNB | 1985 | 152083004 |
| Parque Nacional Torres Del Paine | N315 05111979 MA | 1979 | 152124001 |
| Parque Nacional Vicente Perez Rosales | N369 07031994 - N92 22082017 MBN | 1994 | 152101003 |
| Parque Nacional Villarrica | N2236 28111940 MTC - N2789 29051931 - N398 30061983 MBN | 1940 | 152105005 |
| Parque Nacional Volcán Isluga | N151 261185 MA | 1985 | 152011003 |
| Parque Nacional Yendegaia | N4 27012016 MBN | 2016 | 152121001 |
| Reserva Nacional Alto Bio Bio | N1935 06111912 | 1912 | 152092004 |
| Reserva Nacional Altos de Lircay | N59 110696 MA | 1996 | 152071003 |
| Reserva Nacional Altos De Pemehue | N80 17082009 MBN | 2009 | 152083005 |
| Reserva Nacional China Muerta | N330 28061968 MA | 1968 | 152091005 |
| Reserva Nacional Coihaique | N112 17041980 MBN | 1980 | 152111001 |
| Reserva Nacional El Yali | N41 230596 MA | 1996 | 152056001 |
| Reserva Nacional Federico Albert | N257 250581 MBN | 1981 | 152072001 |
| Reserva Nacional Futaleufú | n602 08091998 MBN | 1998 | 152112001 |
| Reserva Nacional Isla Mocha | N172 30122010 MBN | 2010 | 152082004 |
| Reserva Nacional Katalalixar | N780 21121983 MBN | 1983 | 152124001 |
|  |  |  |  |
| Reserva Nacional La Chimba | N71 120588 MA | 1988 | 152021001 |
| Reserva Nacional Lago Carlota | N204 23061970 MA - N391 08061965 MA - N329 01061967 MA | 1969 | 152111001 |
| Reserva Nacional Lago Las Torres | N632 29091982 MBN | 1982 | 152111001 |
| Reserva Nacional Lago Palena | N159 26021965 MA | 1965 | 152111001 |
| Reserva Nacional Lago Peñuelas | N859 040652 MTC - N284 200870 MA - N7 100185 MBN - N133 280889 MA | 1952 | 152051006 |
| Reserva Nacional Lago Rosselot | N640 13101983 MBN | 1983 | 152112001 |
| Reserva Nacional Laguna De Torca | N128 17101985 MA | 1985 | 152073005 |
| Reserva Nacional Laguna Parrillar | N245 22041977 MA - N423 220682 MBN - N19 11031993 MBN | 1977 | 152121001 |
| Reserva Nacional Las Chinchillas | N153 301183 MA | 1983 | 152042001 |
| Reserva Nacional Las Guaitecas | N420 13071983 MBN | 1983 | 152112001 |
| Reserva Nacional Las Vicuñas | N29 080383 MA | 1983 | 152012001 |
| Reserva Nacional Llanquihue | N883 19101988 MBN | 1988 | 152101003 |
| Reserva Nacional Los Bellotos Del Melado | N18 200195 MA - N43 19122016 MMA | 1995 | 152074003 |
| Reserva Nacional Los Flamencos | N50 020490 MA | 1990 | 152022001 |
| Reserva Nacional Los Huemules Del Niblinto | N32 230299 MA | 1999 | 152084003 |
| Reserva Nacional Los Queules | N12 140395 MA | 1995 | 152072001 |
| Reserva Nacional Los Ruiles | N94 130782 MA - N102 29092017 MBN | 1982 | 152072001 |
| Reserva Nacional Magallanes | N2444 23111939 MT | 1939 | 152121001 |
| Reserva Nacional Malalcahuello | N131 15041987 MBN | 1987 | 152092004 |
| Reserva Nacional Malleco | N1540 30091907 RE - N28 230185 MBN | 1907 | 152083005 |
| Reserva Nacional Mocho Choshuenco | N55 02031994 MA | 1994 | 152105006 |
| Reserva Nacional Nalcas | N604 06101967 MA | 1967 | 152083004 |
| Reserva Nacional Nonguén | N132 30122009 MNB | 2009 | 152081001 |
| Reserva Nacional Ñuble | N384 241178 MA | 1978 | 152084003 |
| Reserva Nacional Pampa Del Tamarugal | N207 181287 - N310 260994 MA - N59 07062013 MBN | 1987 | 152011003 |
| Reserva Nacional Pingüino De Humboldt | N4 030190 MM | 1990 | 152033001 |
| Reserva Nacional Radal Siete Tazas | N89 200396 MBN - N15 27032008 MNB | 2008 | 152073002 |
| Reserva Nacional Río Blanco | N871 141157 MA - N66 100277 MTC | 1932 | 152053001 |
| Reserva Nacional Río Clarillo | N19 29011982 MA | 1982 | 152132002 |
| Reserva Nacional Río Los Cipreses | N127 171085 MA | 1985 | 152063001 |
| Reserva Nacional Río Simpson | N1060 01121999 MBN | 1999 | 152112001 |
| Reserva Nacional Roblería Del Cobre De Loncha | N62 25071996 MA | 1996 | 152135001 |
| Reserva Nacional Trapananda | N357 27081992 MBN | 1992 | 152111001 |
| Reserva Nacional Villarrica | N8129 12081929 MTC | 1929 | 152091012 |

Table S4: Additional robustness estimations. Estimated effects of protected areas on poverty in Chile. The main explanatory variable is a binary variable equal to 1 if the unit has at least a fraction of its area protected.

|  | Outcome of interest: poverty index | | | |
| --- | --- | --- | --- | --- |
|  | (1) | (2) | (3) | (4) |
| Binary variable = 1 if at least 17% is protected | -0.216^*^  (0.127) | -0.213  (0.130) | -0.050  (0.138) | -0.046  (0.142) |
| $D_{it}$* Patagonia, where $D_{it}$is a binary variable = 1 if at least 17% is protected |  |  | -0.580^***^  (0.205) | -0.582^**^  (0.212) |
| $log(population)$ | 0.265  (0.194) |  | 0.263  (0.192) |  |
| Constant | -3.474  (2.311) | -0.378^***^  (0.294) | -3.488  (2.255) | -0.376^***^  (0.029) |
| Unit-fixed effect | Yes | Yes | Yes | Yes |
| Year-fixed effect | Yes | Yes | Yes | Yes |
| No. of observations | 534 | 534 | 534 | 534 |
| $R^{2}$ | 0.914 | 0.913 | 0.916 | 0.915 |

Notes: Robust standard errors clustered at the unit level are reported in parentheses. The levels of significance are ^*^10%, ^**^5%, and ^***^1%.
